# Corrigendum: AGM2015: Antineutrino Global Map 2015

**DOI:** 10.1038/srep15308

**Published:** 2015-10-15

**Authors:** S. M. Usman, G. R. Jocher, S. T. Dye, W. F. McDonough, J. G. Learned

Scientific Reports 5: Article number: 1394510.1038/srep13945; published online: 09012015; updated: 10152015

This Article contains typographical errors where the flux unit ‘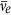/100 cm^2^/s’ was incorrectly given as ‘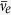/cm^2^/s’

In the legend of Figs 1, 2 and 5,

“Flux units are 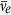/cm^2^/s at the Earth’s surface”

should read:

“Flux units are 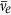/100 cm^2^/s at the Earth’s surface”

In the legend of Fig. 3,

“AGM2015 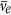 flux (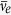/cm^2^/s/keV)”

should read:

“AGM2015 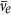 flux (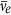/100 cm^2^/s/keV)”

Lastly, in the ‘Geoneutrinos’ section,

“however in this work we focus on simple 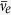 flux (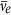/cm^2^/s) and luminosity (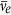/s).”

should read:

“however in this work we focus on simple 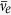 flux (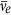/100 cm^2^/s’) and luminosity (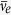/s).”

